# Correction: Challenging the dry core paradigm: hydrated reactivity emerges within micellar cores

**DOI:** 10.1039/d6sc90124g

**Published:** 2026-06-12

**Authors:** Riliga Wu, Tongyue Wu, Weijiang Guan, Chao Lu

**Affiliations:** a State Key Laboratory of Chemical Resource Engineering, Beijing University of Chemical Technology Beijing 100029 China wjguan@mail.buct.edu.cn luchao@mail.buct.edu.cn; b Pingyuan Laboratory, College of Chemistry, Zhengzhou University Zhengzhou 450001 China

## Abstract

Correction for ‘Challenging the dry core paradigm: hydrated reactivity emerges within micellar cores’ by Riliga Wu *et al.*, *Chem. Sci.*, 2026, https://doi.org/10.1039/d6sc03380f.

The authors regret that Fig. 5b and c were mispositioned in the original manuscript. The corrected Fig. 5, along with correct caption text, is detailed below. This does not affect the experimental results, data interpretation, discussion, or conclusions of the article.

**Fig. 1:**
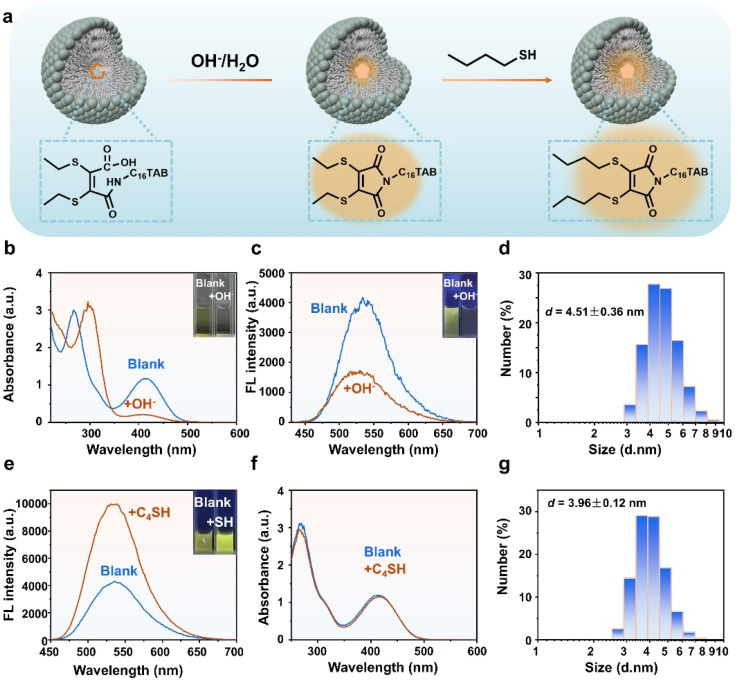
(a) Scheme of the reaction between **DETMI** and hydrophilic hydroxide ions (OH^−^) as well as hydrophobic *n*-butyl mercaptan (C_4_SH). (b) UV-vis spectra, (c) fluorescence emission spectra, and (d) DLS data of **DETMI-C_16_TAB** micelles with the addition of OH^−^. (e) Fluorescence emission spectra, (f) UV-vis spectra, and (g) DLS data of **DETMI-C_16_TAB** micelles with the addition of C_4_SH.

The Royal Society of Chemistry apologises for these errors and any consequent inconvenience to authors and readers.

